# Pulmonary Squamous Cell Carcinoma Dissemination Through Air Spaces (STAS): Clinicopathologic Findings in Different Tumor Origins

**DOI:** 10.3390/pathophysiology33020040

**Published:** 2026-06-17

**Authors:** Bianca Herrmann, Horia Sirbu, Hayk Kikoyan, Mostafa Higaze, Abbas Agaimy, Arndt Hartmann, Ralf Rieker, Mohamed Anwar Haj Khalaf

**Affiliations:** 1 Friedrich-Alexander University Erlangen-Nürnberg (FAU), 91054 Erlangen, Germany; 2 Division of Thoracic Surgery, Erlangen University Hospital, 91054 Erlangen, Germany; 3 Pathology Institute, Erlangen University Hospital, 91054 Erlangen, Germany

**Keywords:** pulmonary squamous cell carcinomas, spread through air spaces, disease-free survival, overall survival

## Abstract

**Background**: Spread through air spaces (STAS) is a recognized histologic pattern of invasion associated with poor prognosis in non-small-cell lung cancer (NSCLC), particularly adenocarcinoma. However, its presence in pulmonary squamous cell carcinoma (SCC), whether primary or metastatic, remains largely unexplored. Given the limited available evidence, this study was designed as an exploratory analysis to evaluate the prevalence and potential prognostic significance of STAS in pulmonary SCC. **Material and Methods**: In this exploratory retrospective study, we analyzed 57 patients who underwent surgical resection for pulmonary squamous cell carcinoma (P-SCC) at the Department of Thoracic Surgery at the University Hospital Erlangen between 2008 and 2020. The cohort included both primary lung SCC and metastatic SCC to the lung from extrapulmonary sites, primarily from ear, nose, and throat (ENT) tumors. Histological slides were reviewed to assess the presence of STAS, as defined by established morphological criteria. The Chi-square test was used to investigate the presence of STAS. Disease-free survival (DFS) and overall survival (OS) was evaluated using Kaplan–Meier analysis, and the prognostic impact of STAS along other variables were assessed using Cox proportional hazards regression. **Results**: A total of 57 patients with squamous cell carcinoma (SCC), 22 (39%) had primary lung SCC and 35 (61%) had metastatic SCC from head and neck tumours (ENT). Spread through air spaces (STAS) was detected in 20 patients (35%). Disease-free survival (DFS) differed according to primary tumour location (*p*-value of 0.009), with higher 1-, 3-, and 5-year DFS in patients with primary lung SCC (86.4%, 77.3%, 63.3%) than in those with head and neck SCC (54.3%, 31.4%, 22.2%). DFS was also significantly higher in patients undergoing solitary resections compared with multiple resections (78.6%, 64.3%, 49.5% vs. 33.3%, 6.7%, not estimable; *p*-value < 0.001). DFS was slightly longer in STAS-negative patients compared with STAS-positive patients (1-, 3-, 5-year DFS: 64.9%, 51.4%, 40.5% vs. 70%, 45%, not estimable), (median DFS 36 vs. 25 months; *p*-value of 0.776). Overall survival (OS) was significantly longer in patients with primary lung SCC (median OS 125 months) than in those with head and neck SCC (27 months; *p*-value of 0.039). STAS-negative patients had also a longer OS than STAS-positive patients (median OS 46 vs. 38 months; HR = 1.11, 95% CI 0.56–2.20; *p*-value of 0.771). **Conclusions:** STAS was identified in metastatic pulmonary SCC lesions as well as in primary lung SCC, occurring in approximately one-third of cases. However, due to the limited cohort size and the exploratory univariate design of the study, the prognostic significance of STAS could not be definitively established and requires further investigation in larger, adequately powered studies.

## Simple Summary

Lung tumors can either originate in the lung or be metastases from other primary sites, such as the head and neck region. “Spread through air spaces” (STAS) represents a specific pattern of cancer-cell spread through the alveolar space and has been associated with poor survival in other types of lung cancer, but its role in squamous cell cancer (SCC) remains unclear. In this study, we analyzed tissue samples from 57 patients with squamous cell cancer and examined the existence of STAS and its impact on recurrence rates and survival. We found that STAS was present in one-third of our cases but had no significant correlation with poorer survival outcome. Our results confirm that STAS may occur in SCC, but its clinical significance remains unknown. This may encourage further studies to explore whether the presence of STAS should be considered in treatment decisions for SCC.

## 1. Introduction

Approximately 85–90% of lung carcinomas are classified as non-small-cell lung cancer (NSCLC) and adenocarcinomas account for the majority of NSCLC cases, about 45% overall, whereas squamous cell carcinomas (SCCs) are less common (20%), based on 2020 data [1]. In addition to primary lung squamous cell carcinomas (SCCs), metastases can also occur in the lungs originating from tumors in other parts of the body. These include SCC of the head and neck region, of which approximately 6% of patients develop pulmonary metastases [2]. In 2015, the world health organization (WHO) introduced Spread through air spaces (STAS) as a new, fourth criterion for tumor invasion. Initially identified in histological slides of adenocarcinomas, it was recognized as a significant prognostic factor for assessing patient outcomes [3]. STAS is associated with increased tumor invasion, more aggressive behavior, and a higher risk of recurrence, all of which have direct implications for therapeutic decision-making, such as the type of resection or the need for adjuvant therapy [4]. Recent studies have identified STAS in approximately one-third of resected pulmonary SCCs, where it independently predicts recurrence and poorer 5-year survival [4,5]. However, its relevance in pulmonary SCC of metastatic origin in various surgical contexts has not been researched [6]. Most studies have focused on primary lung squamous cell carcinoma, whereas metastatic SCC, particularly of head and neck origin, has not been examined [7]. Given the limited available evidence in the literature, the present study was designed as an exploratory analysis to evaluate the prevalence and potential prognostic significance of STAS in pulmonary SCC of both primary and metastatic origin. Due to the relatively small cohort size, the findings should be considered hypothesis-generating and warrant validation in larger studies.

## 2. Materials and Methods

Sample size and eligibility criteria: We conducted a retrospective cohort study that included all consecutive eligible cases meeting our predefined criteria during the study period from 2008 through December 2020. This study includes all eligible patients treated in routine practice during the study period, producing a real-world cohort whose size was determined by case availability rather than by pre-specified statistical power calculations. Before data extraction, we established explicit inclusion criteria: (1) resected primary pulmonary squamous cell carcinoma or pulmonary metastases from SCC of the head and neck region with curative intent; (2) availability of a formalin-fixed, paraffin-embedded specimen with a well-preserved tumor–lung interface suitable for STAS assessment; and (3) complete clinical follow-up data for each patient. Adjuvant treatment consisted of radio chemotherapy or radiotherapy when applicable. Exclusion criteria were: (1) cases without an evaluable tumor parenchyma interface (crushed artifacts, incomplete slides); (2) inadequate or missing histologic material; (3) incomplete clinical or survival data; and (4) prior local or systemic treatments of the tumors that may alter specimen architecture.

Patient study cohort: A 57-patient cohort who underwent surgical resection for primary lung squamous cell carcinoma and for pulmonary metastases of ear, nose, and throat origin at the Department of Thoracic Surgery, University Hospital Erlangen. Because of the rarity of the investigated patient subgroup, the inclusion period was subsequently extended until December 2020 in order to maximize the number of eligible cases. A minimum follow-up of 5 years was required to calculate survival outcomes, which further limited the number of patients available for analysis. Although the study period began in 2008, during which systematic data collection was still being optimized, several early cases could not be reliably included. The index date for overall survival (OS) and disease-free survival (DFS) was defined as the date of definitive lung resection. Overall survival was calculated from this date until death from any cause, whereas disease-free survival was defined as the time from surgery to the first documented recurrence or death.

**Figure 1:**
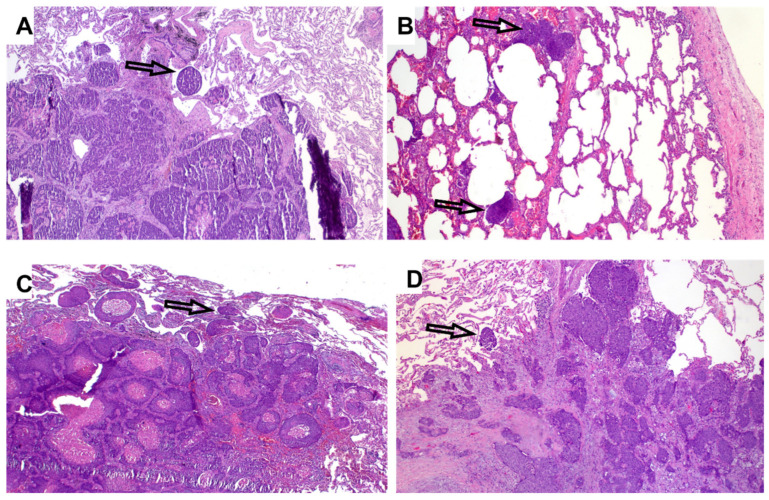
Histopathological findings of squamous cell carcinoma (SCC) with spread through air spaces (STAS). (**A**,**D**) Lung metastasis with STAS clusters (arrowheads) detected in the alveolar spaces of the lung parenchyma beyond the margin of the main tumor. (**B**) Primary pulmonary SCC with intra-alveolar hemorrhage and STAS. (**C**) Primary pulmonary SCC without STAS. Hematoxylin and eosin (H&E) staining; images obtained at ×20 magnification using a Leitz Laborlux 12 light microscope (Leitz, Wetzlar, Germany) equipped with a digital camera.

Histopathological examination: The distinction between primary pulmonary squamous cell carcinoma and pulmonary metastases from head and neck SCC was based on clinical history, prior oncologic diagnosis, radiologic findings, and multidisciplinary tumor board assessment. Histopathology alone was not considered sufficient for definitive classification. Tumor staging at the time of initial diagnosis of the primary tumor was determined according to TNM classification system. Regarding the assessment of STAS, the histopathology slides of the resected pulmonary SCC were independently assessed by two board-certified thoracic pathologists using predefined morphologic criteria for STAS identification. Initial agreement was observed in 52 of 57 cases (observed agreement *P*o = 52/57 = 0.912). Among the concordant cases, 18 were classified as STAS-positive and 34 as STAS-negative. Five cases were discordant and underwent joint review for final adjudication. The expected agreement by chance was calculated, and Cohen’s κ was 0.84, indicating very good interobserver agreement. STAS were first described as a so-called tumor island in 2013 by Onozato et al. [3], and were more precisely defined by the WHO as “micropapillary clusters, solid nests, or single cells beyond the edge of the tumor”, initially referring to adenocarcinomas. These tumor cells are found isolated and without any attachment to the primary tumor or the metastasis in the alveolar tissue [8]. Furthermore, according to Warth et al., these cellular clusters must consist of at least five tumor cells. They can be classified based on their alveolar distance from the primary tumor into limited STAS (<3 alveoli) and extensive STAS (≥3 alveoli) [3]. Given the heterogeneous criteria used to define STAS in previous studies, we defined STAS morphologically as the presence of tumor cells that have no direct connection to the main tumor mass. To minimize the risk of including artifactual cells introduced during metastasis resection or sample handling, STAS was defined as a cluster of more than five tumor cells within the alveolar spaces [9,10]. Additionally, we classified isolated, large clusters of tumor cells (tumor islands) as a distinct subtype of STAS. About the distance parameter, STAS was present in the intra-alveolar spaces within 0.5 mm of the main tumor margin. For example, in cases with collapsed, hemorrhagic, or lymphocyte-infiltrated alveoli, STAS was assessed starting with the third alveolar space adjacent to the main tumor to standardize distance measurement [11] (See Figure 1). Histological assessment was performed using a Leitz Laborlux 12 light microscope (Leitz, Wetzlar, Germany) equipped with a camera scanner and objective lenses of ×10, ×20, and ×40 magnification. Representative histological images were acquired using standard brightfield optics (See Figure 1).

Statistical analysis: The distribution of continuous variables was assessed using the Shapiro–Wilk test. Categorical variables were summarized as frequencies and percentages. Associations between STAS and clinicopathological variables were analyzed using the chi-square test for categorical variables, and the Mann–Whitney U test for non-parametric continuous variables. Survival outcomes were evaluated using the Kaplan–Meier method. Univariable Cox proportional hazards regression analysis was performed to evaluate the association between clinicopathological variables and survival outcomes. Survival analyses were performed using univariate methods only; therefore, the observed associations between STAS and clinical outcomes should be interpreted cautiously, particularly regarding potential confounding clinicopathological variables. The prognostic significance of STAS was further assessed using Cox proportional hazards regression. All statistical analyses were performed using BM SPSS Statistics for Windows, Version 30.0 (IBM Corp., Armonk, NY, USA), with a *p*-value < 0.05 considered statistically significant.

## 3. Results

### 3.1. Patients’ Characteristics

A total of 57 patients were included, comprising 47 males (82%) and 10 females (18%). Most patients were aged ≥65 years (n = 43, 75%). Primary squamous cell carcinoma of the lung was present in 39% of patients (n = 22), whereas 61% (n = 35) were metastases originating from the head and neck region. Adjuvant treatment was administered in 81% of cases, either as radio-chemotherapy or radiotherapy. Preoperatively, all patients were classified according to the American Society of Anesthesiologists (ASA) classification system as ASA class II or III, with ASA III being the most common class in 79% (n = 45). Adjuvant therapy (radio-chemotherapy or radiotherapy) was administered in 81% (n = 46). Anatomical resections were performed in 53% (n = 30) and wedge resections in 47% (n = 27). Most patients underwent solitary resections (n = 42, 74%), while multiple resections were performed in 26% (n = 15). STAS was present at 35% (n = 20) and absent at 65% (n = 37). Lymph node involvement was detected in 35% (n = 20), whereas 65% (n = 37) had negative lymph nodes. Further clinicopathological characteristics are summarized in Table 1.

### 3.2. Distribution of STAS and Association with Clinical Variables

STAS was detected in 20 of the 57 patients examined (35%). Table 2 summarizes the distribution and statistical significance of STAS occurrence across different subgroups. As summarized in the table, no significant associations were observed between STAS and clinicopathological variables (including gender, age, type of resection, number of resections, lymph node involvement, and other variables). Additionally, STAS occurrence was comparable according to tumor origin (primary lung SCC vs. pulmonary metastatic SCC; *p*-value of 0.68), with 35% of STAS-positive and 40,4% of STAS-negative patients having primary pulmonary SCC, and 65% and 59,6% having head and neck SCC, respectively. Since adjuvant treatment does not affect histological assessment of STAS postoperative, the results are reported for the sake of completeness of the table (see Table 1).

### 3.3. Disease-Free Survival (DFS)

Disease-free survival (DFS) was analysed in all patients with a median follow-up of 77 months (IQR: 63–125). The 1-, 3-, and 5-year DFS rates were estimated for each subgroup.

**Figure 2:**
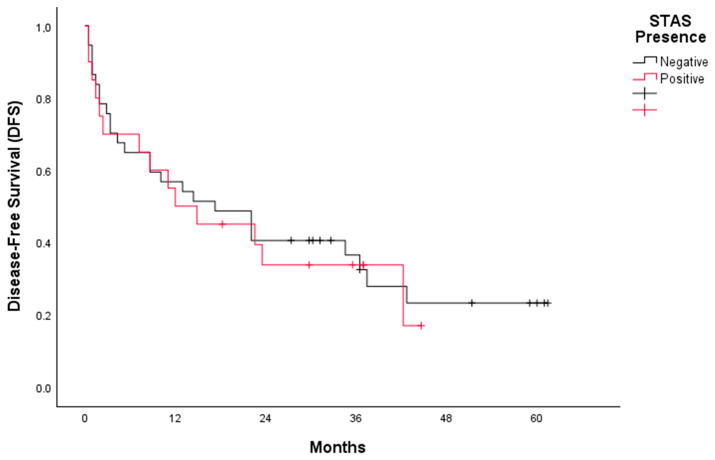
Kaplan–Meier curves for disease-free survival (DFS) according to STAS presence (STAS-positive vs. STAS-negative).

A significant difference in DFS was observed according to primary tumor location (*p*-value of 0.009). Patients with primary lung SCC showed higher 1-, 3-, and 5-year DFS rates (86.4%, 77.3%, and 63.3%) compared with patients with head and neck SCC (54.3%, 31.4%, and 22.2%). Similarly, patients undergoing solitary resections had significantly higher DFS rates (78.6%, 64.3%, and 49.5%) compared with those undergoing multiple resections (33.3%, 6.7%, and not estimable; *p* < 0.001). No statistically significant differences in DFS were observed for gender, age, tobacco consumption, ASA class, adjuvant treatment of the tumor, lymph node invasion, or STAS status (Figure 2). Table 2 summarizes DFS outcomes according to patient variables.

### 3.4. Overall Survival Results

**Figure 3:**
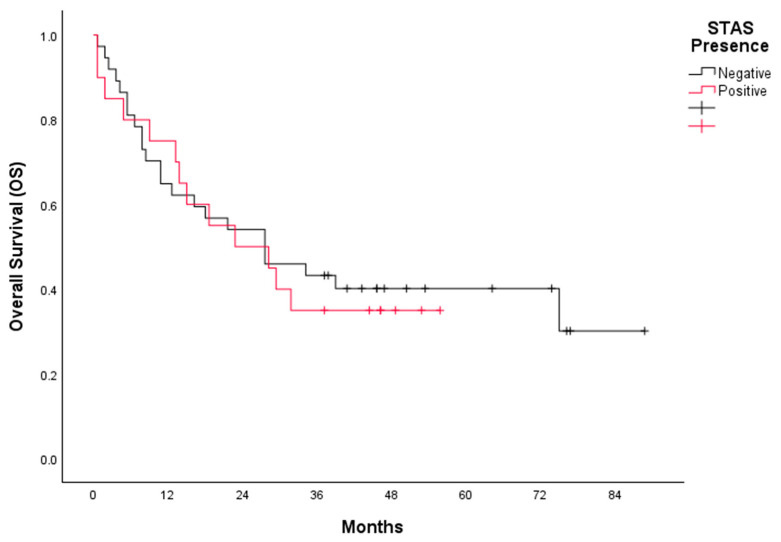
Kaplan–Meier curves for of overall survival (OS) according to STAS presence (STAS-positive vs. STAS-negative).

Overall survival (OS) differed significantly according to primary tumor location. Patients with pulmonary SCC showed a higher survival rate, with a median OS of 125 months, compared with patients with head and neck SCC, who had a median OS of 27 months (*p*-value of 0.039). No significant differences in OS were observed for the other clinicopathological variables analysed using log-rank testing and Cox regression. Comparable overall survival between STAS-positive and STAS-negative patients is illustrated by the Kaplan–Meier curves (Figure 3). Further results are summarized in Table 3.

## 4. Discussion

Including both primary lung tumors and pulmonary metastases from head and neck SCC, STAS was identified in approximately 35% of cases. No statistically significant associations were observed between STAS and other clinicopathological parameters. From a clinical and oncological perspective, patients with primary pulmonary SCC demonstrated significantly higher disease-free survival (DFS) rates compared with patients with metastatic head and neck SCC (*p* = 0.009). Similarly, solitary resections were associated with significantly better DFS compared with multiple resections (*p* < 0.001). However, these findings should be interpreted cautiously, as primary lung SCC is often surgically treated at earlier disease stages, whereas pulmonary metastases from head and neck SCC typically represent recurrent systemic disease occurring after a prior disease-free interval [8]. Consequently, the observed survival difference between primary pulmonary SCC and pulmonary metastases from head and neck SCC is not unexpected. From histopathological point of view, primary pulmonary SCC accounted for 35% of STAS-positive tumors and 40.4% of STAS-negative tumors, while head and neck SCC represented 65% and 59.6% of cases, respectively (*p*-value 0.68). The biological significance of STAS remains a subject of ongoing debate. Because lung tissue is inherently composed of open alveolar spaces, pulmonary specimens are particularly susceptible to mechanical displacement of tumor cell clusters during surgical manipulation and histopathological processing [12,13]. This phenomenon, referred to as Spread Through a Knife Surface, may result in artifactual dissemination of tumor fragments and has raised concerns regarding the interpretation of STAS as a true invasive pattern [12]. The risk of such artifacts may be especially relevant in pulmonary SCC, which often exhibits reduced intercellular cohesion and a greater propensity for cellular detachment [14]. Conversely, accumulating evidence supports the concept that STAS represents a genuine biological phenomenon rather than solely a processing artifact [11,15]. Recent molecular and histopathological studies suggest that a tumor microenvironment characterized by stromal activation, immune cell infiltration, and extracellular matrix remodeling facilitates the detachment and migration of tumor cells into surrounding alveolar spaces [6,16]. In a study using next-generation sequencing, STAS was associated with altered tumor microenvironment properties in SCC, including expression changes related to epithelial–mesenchymal transition and proliferation [16].

Limitations and future prospects: We acknowledge that the relatively small cohort size limits the statistical power of this study and increases the risk of type II error [17]. However, the primary aim of this work was exploratory and hypothesis-generating, focusing on the histomorphological presence and behavior of STAS across pulmonary squamous cell carcinomas, including both primary lung SCC and metastatic head and neck SCC involving the lung [5,13]. Given the rarity of these overlapping clinical scenarios, assembling larger single-center cohorts remains challenging. To ensure methodological rigor despite the limited sample size, strict pathological review criteria were applied, including joint multi-head microscope adjudication in discordant cases to minimize interpretation artifacts and artificial tumor displacement. The inclusion of both primary pulmonary squamous cell carcinoma and pulmonary metastases from head and neck SCC introduces differences in the oncologic stage, which may influence survival outcomes independently of STAS [18]. Although primary pulmonary SCC and metastatic HNSCC represent clinically distinct entities, the aim of this exploratory pilot study was to investigate STAS as a shared histomorphological phenomenon of SCC within the pulmonary microenvironment, independent of anatomical origin [19]. In many cases, distinguishing primary pulmonary SCC from metastatic SCC relies largely on clinical correlation and exclusion of extrapulmonary SCC sites rather than absolute histopathological differences alone [20]. Nevertheless, the heterogeneity of the pooled cohort remains a major limitation, and the findings should be interpreted cautiously.

Because this study included all consecutive cases meeting predefined inclusion and exclusion criteria, no prospective sample size calculation was performed. The lack of standardized criteria for identifying STAS may also limit the reliability and comparability of the results [21]. Furthermore, differentiation between “de novo STAS” and artifacts of tissue preparation remains a recognized histopathological challenge, as displaced tumor cells can mimic STAS but typically have a more regular incidence along the resection margin [14,22]. Future studies should prospectively record independent ratings to allow complete reproducibility analysis with the need for future quantitative and morphology-oriented STAS classification systems [23]. As a potential selection bias, it must be acknowledged that only resected tumors were included in this study cohort. So, patients with more advanced tumor stages or irresectable tumors are not represented in the statistics, which may limit the universality of our findings. Additionally, the availability and general quality of achievable tissue samples may have further influence on our selection bias.

Despite these limitations, to our knowledge, this work is the first to consider pulmonary metastatic SCCs of ENT origin when examining the prognostic significance of STAS. Although our results do not confirm a definitive prognostic role, the observed trends regarding tumor origin and resection type suggest a chance of clinical relevance.

Future research should focus on prospective, multicenter studies with larger and more homogeneous cohorts to validate the prognostic relevance of STAS in both primary and metastatic pulmonary squamous cell carcinoma and to clarify whether the presence of STAS should influence surgical decision-making and adjuvant treatment. Centralized pathological review and standardized STAS assessment protocols may further improve reproducibility. In addition, tumor-specific analyses of pulmonary metastases are warranted. In this context, we have recently published data on colorectal cancer lung metastases, and ongoing studies are evaluating pulmonary metastases from sarcoma, which may provide complementary insights into the role of STAS across different metastatic entities.

## 5. Conclusions

We identified STAS in pulmonary SCC independent of tumor origin. However, given the exploratory nature of this study, the limited cohort size, and the restricted number of clinical events, reliable multivariable adjustment for potential confounding factors such as tumor stage, extent of resection, and treatment-related variables was not feasible. Therefore, the present analysis should primarily be considered a hypothesis-generating investigation into the histomorphological presence and behavior of STAS across pulmonary squamous cell carcinomas rather than a definitive prognostic model.

## Figures and Tables

**Table 1:** Comparison of clinicopathological characteristics between STAS-positive and STAS-negative patients. Categorical variables are presented as numbers and percentages. Differences between groups were analyzed using the chi-square test, and corresponding *p*-values are reported.

Variable	Category	n = 57	Presence of STAS		*p*-Value
			STAS-Positive (n = 20)	STAS-Negative (n = 37)	
Gender	Male	47 (82%)	19 (95.0%)	28 (75.7%)	0.07
	Female	10 (18%)	1 (5.0%)	9 (24.3%)	
Age	<65	14 (25%)	5 (25%)	9 (24.3%)	0.95
	≥65	43 (75%)	15 (75%)	28 (75.7%)	
ASA Classification	2	12 (21%)	5 (25%)	7 (18.9%)	0.59
	3	45 (79%)	15 (75%)	30 (81.1%)	
Tabaco consumption	No	26 (46%)	11 (55%)	15 (40.4%)	0.30
	Yes	31 (54%)	9 (45%)	22 (59.6%)	
SCC primary location	Lung	22 (39%)	7 (35%)	15 (40.4%)	0.68
	Head & Neck	35 (61%)	13 (65%)	22 (59.6%)	
Type of resection	Anatomical	30 (53%)	10 (50.0%)	20 (54.1%)	0.77
	Wedge	27 (47%)	10 (50.0%)	17 (45.9%)	
Number of resections	Solitary	42 (74%)	15 (75%)	27 (73%)	0.87
	Multiple	15 (26%)	5 (25%)	10 (27%)	
Lymph Node Status (N)	Positive	20 (35%)	8 (40%)	12 (32.4%)	0.57
	Negative	37 (65%)	12 (60%)	25 (67.6%)	
Pulmonary Tumor size (T)	T1–T2	38 (66.6%)	15 (75%)	23 (62.2%)	0.33
	T3–T4	19 (33.4%)	5 (25%)	14 (37.8%)	
Adjuvant radio-chemotherapy/radiotherapy	Yes	46 (81%)	17 (85%)	29 (78.4%)	0.55
	No	11 (19%)	3 (15%)	8 (21.6%)	

**Table 2:** Disease-free survival (DFS) according to clinicopathological variables. DFS rates at 1, 3, and 5 years were estimated using the Kaplan–Meier method. Differences between groups were assessed using the log-rank test. Median follow-up (FU) is presented as months with interquartile range (IQR). Abbreviations: DFS, disease-free survival; FU, follow-up; IQR, interquartile range; CI, confidence intervals.

Variable	Group	n	Events	Censored	Median FU–DFS Months (IQR)	1-Year DFS %	3-Year DFS %	5-Year DFS %	Median DFS Months (95% CI)	Log–Rank *p*
Gender	Male	47	33	14	77 (63–123)	68.1	46.8	37.9	31 (7.9–54.1)	0.757
	Female	10	7	3	107 (68–125)	60	50	40	18 (0.0–75.3)	
Age	<65	14	11	3	93 (63–107)	64.3	28.6	21.4	18 (12.5–23.4)	0.36
	≥65	43	29	14	77 (65–125)	67.4	55.8	43.8	46 (22.3–69.6)	
Tabaco consumption	Yes	31	20	11	77 (62–93)	80.6	64.	47.7	11 (0.0–23.9)	0.111
	No	26	20	6	107 (76–125)	50	30.8	26.9	49 (12.6–85.3)	
ASA Classification	2	12	9	3	123 (93–125)	75	66.7	50	46 (0.0–101.4)	0.52
	3	45	31	14	74 (63–107	64.4	44.4	35.2	25 (7.9–42)	
SCC primary location	Lung	22	12	10	77 (65–125)	87	75.4	62.6	18 (5.3–30.6)	0.009
	Head & Neck	35	28	7	93 (76–107)	53.0	32	22	76 (67.9–84)	
Lymph Node Status	Negative	37	25	12	107(68–125)	70.3	54.1	45.9	46 (0–95.5)	0.306
	Positive	20	15	5	77 (65–93)	60	40	23.3	18 (0.4–35.5)	
Type of Resection	Anatomical	30	21	9	77 (62–125)	70	53.3	40	69 (5.1–66.8)	0.871
	Wedge	27	19	8	77 (74–107)	63	44.4	36.4	70 (7.9–52)	
Number of Resections	Solitary	42	25	17	77 (65–123)	78.6	64.3	49.5	49 (18.1–79.8)	<0.001
	Multiple	15	15	0	-	33.3	6.7	–	4 (1.5–6.4)	
Adjuvant radio-chemotherapy/radiotherapy	Yes	46	32	14	125 (77–125)	63	45.7	36.6	25 (5–44.9)	0.765
	No	11	8	3	77 (63–123)	72.7	54.5	45.5	49 (0.1–97)	
STAS Presence	Negative	37	26	11	107 (65–125)	64.9	51.4	40.5	36 (11.1–60.8)	0.776
	Positive	20	14	6	77 (62–93)	70	45	–	25 (7.4–42.1)	

**Table 3:** Comparison Overall survival (OS) according to clinicopathological variables. Median OS was estimated using the Kaplan–Meier method. Differences between groups were assessed using the log-rank test. Hazard ratios (HRs) with 95% confidence intervals (CIs) were calculated using univariable Cox proportional hazards regression analysis. Female sex, age ≤ 65 years, non-smoker status, primary lung SCC, ASA class < 3, wedge resection, lower tumour stage, no adjuvant radio-chemotherapy/radiotherapy (RCT/RT), negative lymph node status, and STAS-negative tumours were used as reference categories. Abbreviations: OS, overall survival; HR, hazard ratio; CI, confidence interval.

Variable	Group	N	Events	Censored	Median OS Months (95% CI)	Log–Rank *p*	HR (95% CI)	*p* Value
Gender	Male	47	30	17	46 (0.0–137)	0.674	1.208 (0.499–2.925)	0.676
	Female	10	6	4	46 (24.5–67.4)			
Age	<65	14	10	4	27 (10.9–43–5)	0.403	0.733 (0.351–1.53)	0.408
	≥65	43	26	17	47 (22.5–71.4)			
Tabaco consumption	Yes	31	18	8	23 (8–37.9)	0.282	0.700 (0.362–1.352)	0.288
	No	26	18	13	49 (25.5–72.4)			
ASA Classification	2	12	7	5	53 (14.6–91.3)	0.455	1.366 (0.597–3.125)	0.459
	3	45	29	16	36 (8.6–63.6)			
SCC primary location	Lung	22	11	11	125 (43.3–206.6)	0.042	0.461 (0.221–0.961)	0.039
	Head & Neck	35	25	10	27 (28–64.9)			
Lymph Node Status (N)	Negative	37	20	17	57 (17.7–96.2)	0.049	1.918 (0.988–3.721)	0.054
	Positive	20	16	4	23 (0.0–49)			
Type of resection	Anatomical	30	19	11	46 (17.8–74.1)	0.753	0.901 (0.467–1.736)	0.754
	Wedge	27	17	10	38 (10.8–65.1)			
Number of Resections	Solitary	42	25	17	47 (23.1–70.8)	0.196	0.630 (0.309–1.284)	0.203
	Multiple	15	11	4	23 (1.5–44.4)			
Adjuvant radio-chemotherapy/radiotherapy	Yes	46	29	17	38 (19.1–56.7)	0.745	1.146 (0.502–2.617)	0.747
	No	11	7	4	49 (3.6–94.3)			
STAS presence	Negative	37	23	14	46 (13.9–78)	0.776	1.107(0.557–2.199)	0.771
	Positive	20	13	7	38 (2.9–46)			

## Data Availability

The study data are not publicly available due to privacy and ethical restrictions. The data may be available from the corresponding author upon reasonable request and subject to approval by the responsible institutional authorities.

## Abbreviations

The following abbreviations are used in this manuscript:

STAS	Spread through air spaces
SCC	Squamous cell carcinoma
P-SCC	Pulmonary squamous cell carcinoma
NSCLC	Non-small-cell lung carcinoma
ENT	Ear nose and throat
OS	Overall survival
DFS	Disease-free survival
